# Direct Infusion Metabolomics of the Photosystem and Chlorophyll Related Metabolites within a Drought Tolerant Plant Introduction of *Glycine max*

**DOI:** 10.3390/metabo11120843

**Published:** 2021-12-06

**Authors:** Kevin J. Zemaitis, Heng Ye, Henry T. Nguyen, Troy D. Wood

**Affiliations:** 1 Department of Chemistry, Natural Sciences Complex, University at Buffalo, State University of New York, Buffalo, NY 14260, USA; kevinzem@buffalo.edu; 2 Division of Plant Sciences and National Center for Soybean Biotechnology, University of Missouri, Columbia, MO 65211, USA; yehe@missouri.edu (H.Y.); nguyenhenry@missouri.edu (H.T.N.)

**Keywords:** plant introduction, drought stress response, slow wilting canopy, plant growth, drought tolerance, chlorophyll content, phytochemicals, soybean, direct infusion, FT-ICR, metabolomics

## Abstract

Drought is the most prolific form of abiotic stress that legumes and cereal plants alike can endure, and the planting of an improper cultivar at the beginning of a season can cause unexpected losses up to fifty percent under water deficient conditions. Herein, a plant introduction (PI) of an exotic cultivar of soybean (*Glycine max*), PI 567731, which demonstrates a slow wilting (SW) canopy phenotype in maturity group III, was profiled under drought conditions in field trials in Missouri against a drought susceptible check cultivar, Pana. Metabolomic profiling was carried out on samples of leaves from each of these cultivars at V5 and R2 growth stages both while irrigated and while under drought stress for three weeks. PI 567731 was observed to have differential phytochemical content, and enhanced levels of chlorophyll (Chl) a/b and pheophytin (Pheo) were profiled by direct infusion electrospray Fourier transform ion cyclotron resonance mass spectrometry (FT-ICR MS). Indicating drought induced changes of the photosystem and photosynthetic capabilities alongside water preservation strategies are important within the SW phenotype drought response. Subsequent multivariate analysis was able to form predictive models, encompassing the variance of growth and drought stress of the cultivar. Moreover, the existence of unique Chl-related metabolites (CRM) (*m/z* > 900) were confirmed through tandem mass spectrometry. The resultant coordination of fatty acids to the core of the porphyrin ring was observed and played an unknown role in the proliferation of the photosynthesis. However, the relative ratio of the most abundant CRM is undisturbed by drought stress in PI 567731, in contrast to the drought susceptible cultivar. These results provide key insights into drought related metabolic mechanisms.

## 1. Introduction

Agricultural crops can endure a matrix of stress resulting from a variety of sources including biotic or abiotic stressors such as drought, flooding, and salinity, or nutrient availability [1]. Among the different sources, varying levels of water deficiency and drought have the most prolific and detrimental effect to agricultural farms on the national and global scale [2]. Numerous plant traits have been identified for the potential of improving the performance of drought-affected crops, mainly through conservation of water [3]. However, more recent works identifying the importance of slow canopy wilting (SW) phenotypes for their potential stress tolerance in water deficient environments [4]. Legumes, such as soybean (*Glycine max*), have a particular intolerance to water deficiency in the early stages of growth and flowering, where a decrease in water availability by half can result in up to a loss of half the expected yields [5]. With persistent changes in climate predicted, further detrimental impacts on agriculture in the coming decades are to be expected [6], creating a present need for further understanding of positive and negative water deficient responses.

Recently, an exotic soybean germplasm, plant introduction *(PI) 567731* in maturity group III (MG III), was identified to consistently express the SW phenotype in the field compared to the drought sensitive cultivar Pana [4,7]. A major SW quantitative trait loci (QTL) (*qSW_Gm10*) was mapped on chromosome 10 from PI 567731 through a genetic study in a recombinant inbred line (RIL) population, and this QTL was further confirmed to delay canopy wilting under drought conditions in a near-isogenic background [4]. After identification of this SW QTL, PI 567731 showed lower yield loss than Pana under drought stress with greater than 13% more yield index (yield under rain-fed/yield under irrigation) [7], and was also found to use significantly less water under drought conditions. Thus, a water conservation strategy was identified as being associated with limited-maximum transpiration rates. The transpiration of PI 567731 was also found to be sensitive to an aquaporin inhibitor (silver-nitrate), indicating the independence of a limited-maximum transpiration to a lack of silver-sensitive aquaporins in these SW genotypes. Overall, this PI showed great promise within field trials for a positive water deficient response, but many underlying metabolomic mechanisms are not well understood [8].

In efforts to understand observations from field trials and mapping of QTLs, many researchers have adopted the use of mass spectrometry (MS) based proteomic and metabolomic techniques. These platforms and subsequent data analysis pipelines further reinforce and probe the mechanisms of plant stress responses. As positive and negative stress responses are characteristic to either acute or prolonged effects to drought stress, the initial impacts primarily effect net photosynthesis and photosynthetic performance of the plants. Under drought stress, stomatal closures and hormonal signaling through abscisic acid have been identified as the key reductants to net photosynthesis [9]. With increased efficiency of the photosystem (PS) II denoted in positive stress tolerance [10]. Extended periods of drought stress have been demonstrated to also induce reduced chlorophyll (Chl) content and related fluorescence parameters, which are critical in considerations of the photosystem (PS) II [11]. Drought stress has also been noted to induce reordering of the PSII core [12]. Although water deficiencies do not directly impact the primary components of C3 plants PSI or PSII directly, these secondary impacts are well known in a variety of crops to reversibly impact photosynthesis, prior to photosynthetic decay [13]. This emphasizes the need for targeted approaches of profiling phytochemicals as a reliable means of screening for stress tolerances, including drought tolerance [14,15].

Both targeted and non-targeted approaches for determining metabolic profiles of soybean and other agronomical crops have been entailed with instrumental approaches ranging from gas or liquid chromatography (GC/LC) coupled with mass spectrometry (MS) [16,17], nuclear magnetic resonance (NMR) [18,19], and a variety of spectroscopic techniques for in-vivo studies [10]. However, no one all-inclusive method for simultaneous detection of all metabolites is available. With a broad array of expression in a variety of primary and secondary metabolites in model plants and agricultural crops, methods either prove to be either moderate throughput with high specificity in the case of extracts or lack specificity with high-throughput analysis as is the case for spectroscopic measurements. High-resolution accurate mass MS platforms such as Fourier transform ion cyclotron resonance (FT-ICR), due to unprecedented mass resolving power and mass accuracy [20,21], allows for the direct infusion of samples with no on-line separations [22]. In comparison to other MS profiling techniques, direct infusion FT-ICR holds at least a ten-fold decrease in analysis time, while simultaneously detecting hundreds of metabolic signals. As such, the platform is ideal for determination of relative phytochemical content despite several limitations. However, when utilized in tandem with physiological data and other interconnected pathways, insight on the acclimatization of photosynthesis in stress tolerances can be gleaned [23]. Herein described is the targeted profiling of phytochemical content and multivariate analysis of a drought tolerant cultivar, PI 567731, in comparison to a drought susceptible cultivar, Pana, grown in field trials, with drought treatment consisting of no irrigation or rainfall for three weeks.

## 2. Results and Discussion

### 2.1. Multivariate Analysis of Cultivar Treatments

**Figure 1:**
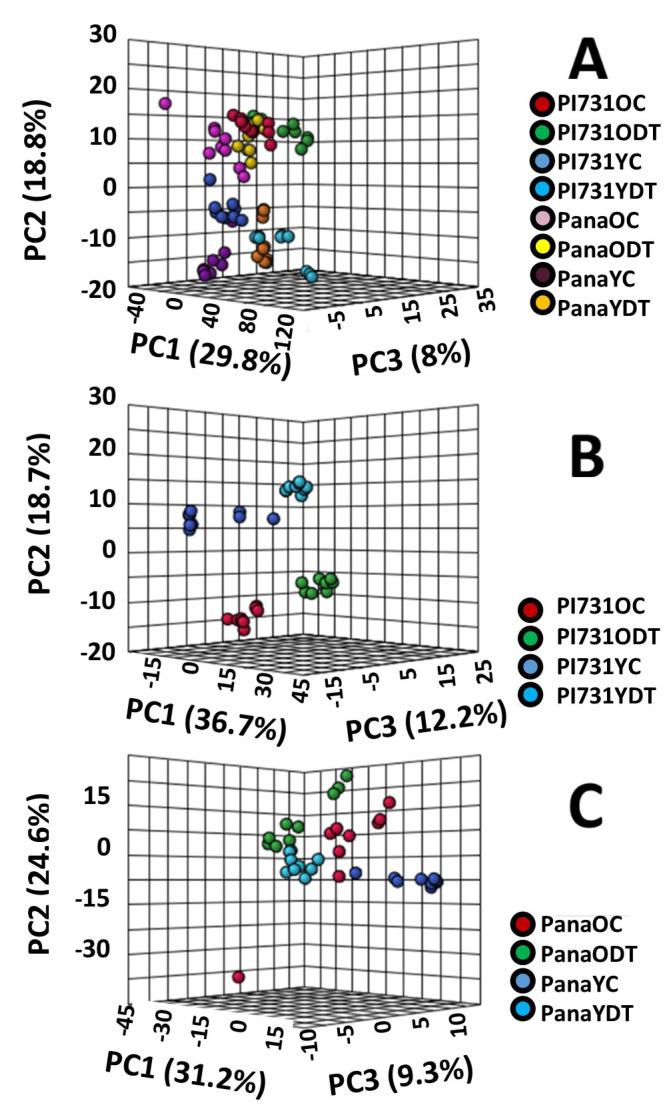
(**A**) is a 3D scores plot of the PCA of all Pana and PI 567731, (**B**) is the 3D−PCA of all PI 567731 datasets, and (**C**) is the 3D−PCA of all Pana datasets. Each legend is unique to each scores plot, and is to the right of each plot.

Principal component analysis (PCA) was performed as an orthogonal model to partial least squares discriminant analysis (PLS-DA) in order to discriminate subsets of drought treatment in the cultivars in MetaboAnalyst 5.0. Shown in Figure 1 are the three-dimensional (3D) PCA of the entire sample population and models segmented according to the cultivar. This was completed to identify outliers initially from the 72 samples with an average of 2560 peaks per sample, after which further analysis was completed. The models demonstrate the distinct variance in the datasets from the control and drought treated metabolic fingerprints, as well as the unique variance to each cultivar, allowing for the distinction amongst the DI FT-ICR MS datasets.

**Figure 2:**
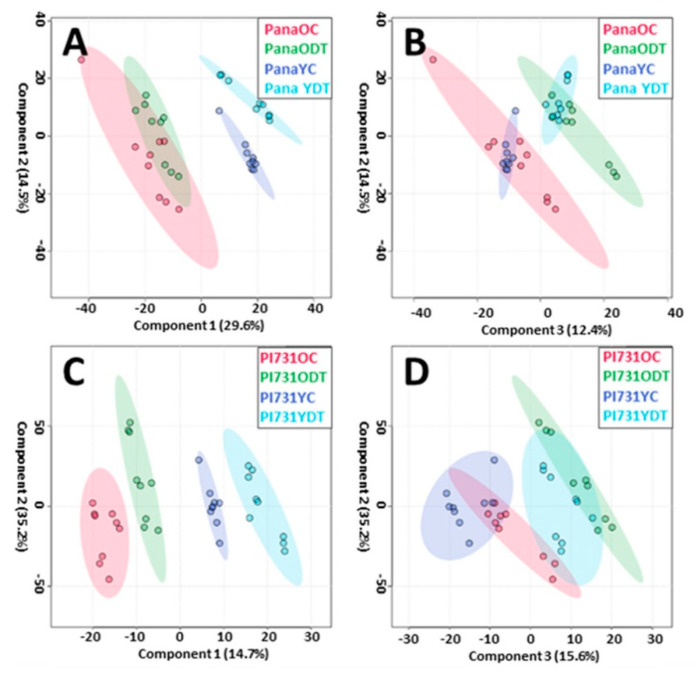
PLS−DA score plots of the first and second components (**A**,**C**) and the second and third components (**B**,**D**) of all subsets of Pana (**A**,**B**) and PI 567731 (**C**,**D**) samples. With a window of 95% confidence around the encompassed variance.

Alongside of the drought treatment, samples of these cultivar were also collected from two separate growth stages, including V5 and R2. The vegetal growth stage was determined by counting of nodes within the main stem of the plant, and reproductive stage based upon the flowering and pod development [24]. As shown in Figure 2A,B within PLS-DA models, the variance of within these separate stages of growth was able to be separated within the matrix of metabolites detected. For the young control (YC) and young drought treated (YDT) samples, identified to be within stage V5 of growth, the first component encompassed variance which separates the datasets, and the third component encompassed the variance imposed by drought itself based upon the groupings. These analyses utilize 95% confidence intervals in the scores plots, in the form of ellipses surrounding each subset population, allowing for the visual investigation of the analysis of variance between models. From the first component of the PLS-DA (Figure 2A,C), Pana datasets within the model had greater variance explained and both cultivars were distinguished by physiological age grouping. The third component of the model (Figure 2B,D) demonstrates overlapping confidence intervals in the case of PI 567731, with less observable variance within the metabolic fingerprint for both YDT/old drought treatment (ODT), having compositions closer to that of the YC/old control (OC) in the third component. PLS-DA is the most implemented tool in metabolomics datasets for a multitude of reasons. However, caution must be utilized with the raw data matrix, as the groupings can lend to a tendency to over fit the model when a variety of factors are not considered [25]. Based upon comparisons to the PCA, and other measures within MetaboAnalyst, this was not observed. So as to not impart effects of growth on the further targeted analysis, OC/ODT were further analyzed independent of the YC/YDT datasets.

### 2.2. Profiling Chlorophyll Content in the Treatments

**Figure 3:**
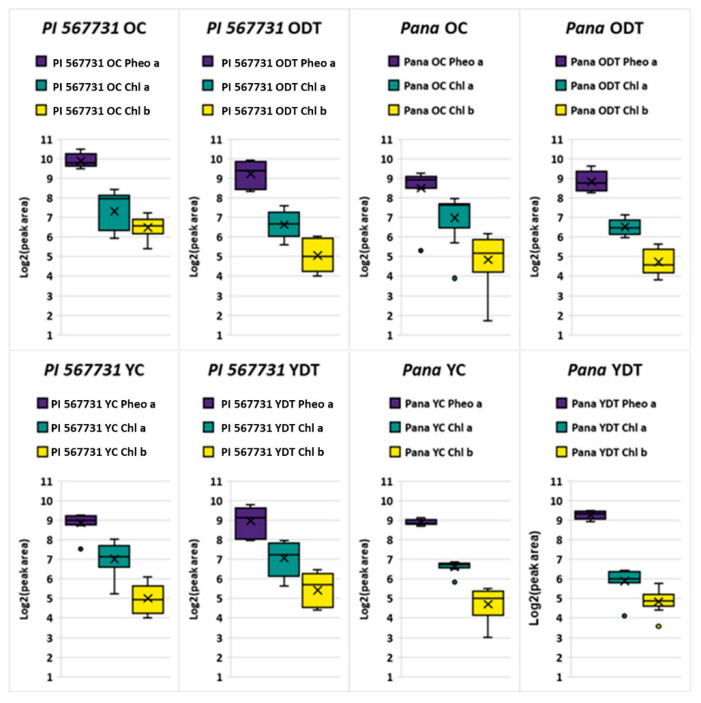
General log scale box and whisker plots of the binned protonated and sodiated adducts from Pana and PI 567731 for Pheo a, and Chl a/b for the OC/ODT and YC/YDT. The legend above each representative plot provides the color for the corresponding cultivar and treatment condition, where the solid black line within the plot represents the median of the peak area, and black X represents the mean peak area for all replicates.

PI 567731 was identified to preserve soil water through limited-maximum transpiration rates; this behavior would ultimately result in increased stomatal closures throughout the plant’s life cycle, and decreased photosynthesis. Electron transport rates, carboxylation rates, respiration rates, and intrinsic water use efficiencies follow the dependencies of increased stomatal closures exhibited in water deficient events, with photosynthetic decay only occurring over extended periods of stress [26,27]. Throughout the profiling of the OC/ODT and YC/YDT datasets, it was observed that increased levels of Chl a/b, and Pheo a were observed to be statistically enhanced components within the samples of PI 567731, with an overall median increase in relative abundance of Pheo a and Chl b detected within PI 567731 as shown in the box and whisker plots in Figure 3. 

T-tests were conducted for individual adducts for treatments of Pana and PI 567731. PI 567731 OC was found to have statistically significant log fold increases (*p* ≤ 0.05) for the sodiated adduct Chl b (*p* = 0.010) and Pheo a (*p* = 0.0006), as well as the protonated Pheo a (*p* = 0.033) in reference to Pana OC. Sodiated Chl b content was found to be significantly enhanced in PI 567731 ODT (*p* = 0.039) in reference to Pana ODT. Distributions for the YC/YDT found significance for the protonated Chl a (*p* = 0.0005) in the YC of PI 567731 and protonated form of Pheo a (*p* = 0.019) in the YDT of PI 567731 over the corresponding Pana OC/ODT trials. Individual adducts account for the log fold distributions within the binned peak areas shown in the OC/ODT and YC/YDT plots in Figure 3, with Pheo a levels remaining consistent and highlights of elevated levels of Chl b for PI 567731.

The average abundances and ratio of these metabolites has previously been demonstrated as secondary links to the holistic health of the plant, as demonstrated in previous works on Chl and related metabolites as markers for stress tolerance [28]. Increased total Chl content was observed to be statistically significant by analysis of variance (ANOVA) with a *p* ≤ 0.05; previously this has been observed within positive chilling tolerance responses in maize [29]. With the noted increased yield index from the PI, alongside the QTL SW phenotypic expression, this increase in total Chl content is indicative that Chl content is up-regulated when the SW MGIII cultivar experiences drought stress, especially in earlier stages of growth. The observable enhancement was maintained under the drought treatment in younger populations of leaves, signifying that the three weeks of drought treatment with subsequent irrigation did not cause degradation. However, it did cause a shift to the average Chl a/b ratios.

The enhanced expression of Chl b relative to Pana also marks proliferation of the photosystem, in agreement with increasing the breadth of the photosynthetic antennae in plants with positive stress responses [30]. The decrease within PI 567731 for Chl b is also indicative of drought stress responses, while still remaining consistent with the drought susceptible control levels of Pana when PI 567731 is under drought stress during later stages of growth. Within studies of maize and rice with known water deficient intolerances, the photosynthetic antenna has been shown to be broadened, and an altered ratio of Chl a/b within tolerant cultivars is observed [31]. It has also been demonstrated Chl b is not just an accessory pigment in the light harvesting system, and can play a more pertinent role in primary light harvesting complexes [30], possibly allowing for more efficient use of light despite limited-maximum transpiration rate within earlier stages of growth. Moreover, the relative content should be at the same level of physiological responses from Pana if there was no existence of a positive stress response enhanced by a broadened photosynthetic antenna. The mapping of the SW QTL on chromosome 10 further suggests that in times of stomatal openings these increased levels of Chl content in the PI allow for effiecient photosynthesis.

### 2.3. Tandem Mass Spectrometry of Novel Chlorophyll Related Metabolites

**Figure 4:**
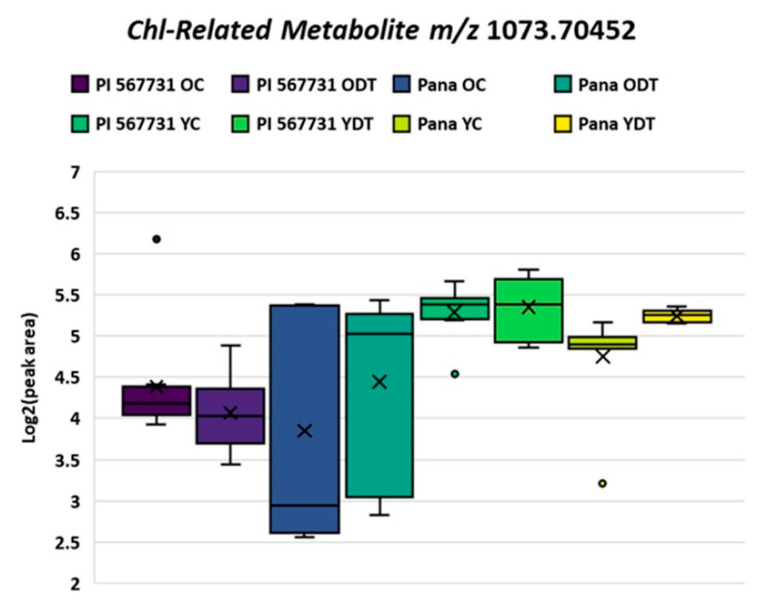
Box and whisker plot of peak area of the CRM at *m/z* 1073.70483 in a relative log scale abundance. The plots represent both Pana and PI 567731 in both OC/ODT and YC/YDT datasets, the legend provides the color for the corresponding cultivar and treatment condition above.

**Figure 5:**
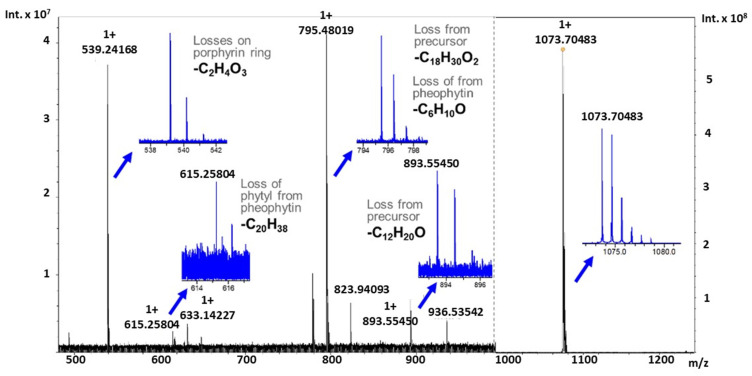
CID of CRMs at *m/z* 1073.70483 at 30 eV with argon collision gas, the precursor y-axis scale is ×10^8^ where fragment ions scale is ×10^7^ Characteristic losses from Pheo a occur with losses highlighted from either the sodiated ion at *m/z* 893.55514 (0.64 ppm error) or the precursor CRM ion at *m/z* 1073.70483 (1.61 ppm error). Zoomed views of the entire isotopic distribution is noted with the molecular losses.

While further profiling the annotated metabolites, distinct novel CRMs previously reported within soybean extracts were also denoted within this research, highlighted by CRM at *m/z* 1073.70740 [32]. As shown in the box and whisker plot in Figure 4, general log fold increases are experienced comparing young to older populations, with an increased median expression in drought treatment of the susceptible cultivar Pana. However, PI 567731 levels of this sodium adduct remain stable through drought stress response. Through the fragmentation of the molecules by collision induced dissociation (CID), these species (>893 Da) showed characteristic losses to that of a porphyrin ring base. Distinct moieties attached directly to the porphyrin ring are apparent by the neutral losses from the precursor ion, which still exhibits characteristic loss of the phytyl group (C_20_H_38_), as shown in the spectrum in Figure 5. 

Once isolated, a product ion at 893.55473 Da forms, corresponding to a sodium adduct of Pheo a through the neutral loss of 180.15031 Da. When a neutral loss search within METLIN is completed with annotation in LIPID MAPS, yielding matches with a conjugated fatty acid (C_12_H_20_O). Upon further fragmentation, neutral losses of 278.29692 Da and 76.01606 Da appear, as annotated in Table 1, corresponding to the loss of the phytyl group (C_20_H_38_) to pheophorbide a, and to the loss of moieties on the porphyrin ring (C_2_H_5_O_3_), respectively. These results are consistent with previously reported literature studies of fragmentation of both Chl a and Pheo a by various dissociation techniques on FT-ICR MS [33,34]. A generation of a neutral loss under weak collisional energies (and the loss of an intact phytyl) suggests a weak coordination or bond formed to the porphyrin ring, not a modification that is not directly linked to the phytyl group. However, fractionation and NMR will be needed to confirm the fragmentation results.

Literature reports have noted increased fatty acid and lipid content within mesophyll membranes and chloroplasts to be essential for various stress tolerances [35], and are postulated to be pertinent to regulation of chloroplasts, especially under low or high temperatures, and abiotic stress [36,37]. This has a further influence upon Chl-protein complexes, with heterogeneous lipid and fatty acid composition of the membranes. With various CRMs (>900 Da) being reported, fragmentation does confirm relation to Chl and Pheo in the porphyrin metabolism for these metabolites due to characteristic neutral losses. However, the metabolic pathway for the attachment and position on the porphyrin ring is unknown. Considering that many of the species were also heavily oxidized, as is apparent by high resolution accurate mass annotation of the full scan mass spectrum, this could also be a byproduct of reactive oxygen signaling (ROS) or of Chl-binding complexes in PSII membranes. Further study of these molecules is warranted for structural elucidation.

## 3. Materials and Methods

### 3.1. Chemicals

Quinapril HCl used was a USP Reference Standard (Rockville, MD, USA). Methanol (HPLC Grade) was from Sigma-Aldrich (St. Louis, MO, USA), and formic acid 88% (Certified ACS) was from Fisher Scientific (Fair Lawn, NJ, USA) Whatmann (Cat. 1001-055) filtration papers were used for vacuum filtration of particulate matter.

### 3.2. Samples Used during the Study

Two cultivars of soybean (*Glycine max*), PI 567731 and Pana, were grown in field trials at the University of Missouri (latitude 38.895305, longitude −92.205917) with three replications planted for each cultivar in twelve-foot lines for both control and drought conditions. The control sets of each cultivar were grown in the field 20 m away from each other under well-watered conditions which consisted of irrigation once a week, with the other two sets of each cultivar experiencing drought conditions with no irrigation and rainfall for three weeks in the field. Sample collection was completed with the control condition irrigated throughout the growth periods and two days prior to sample collection. Leaf samples were collected at two physiological stages (young at V5 growth stage and old at R2 growth stage). After collection, leaves were flash frozen and transported at −80 °C to be stored in polycarbonate petri-dishes at −20 °C until extractions were processed. 

### 3.3. Metabolite Extraction Protocol

The experimental and control groups of each respective growth period and cultivar underwent a pooling of plant tissue from the leaves collected from several different plants. The samples were weighed out individually, and flash frozen with liquid nitrogen prior to maceration. The tissue was then macerated in an aliquot of ethanol solvent for a minute, after which the remaining portion of solvent was added with internal standard. Maceration continued for five minutes and particulate matter was subsequently removed through vacuum filtration. Samples were then dried in a vacuum oven under pressure of 15 inHg at ambient temperatures and diluted to constant volume.

### 3.4. Data Collection and Processing

All spectra were acquired on a dual-source Bruker Daltonics 12T SolariX FT-ICR mass spectrometer (Bremen, Germany) by electrospray ionization (ESI) of the samples. 100 scans were collected for each dataset for multivariate analysis at 2 Megaword (Mw) using broadband detection from *m/z* 147.4 to 1500, resulting in a transient of 0.8389 sec. Solvent extracts were analyzed in triplicate for instrumental replicates (n = 3) and triplicate technical replicates (n = 3) for each growth stage of the two cultivars for both experimental conditions. Spectra were processed in DataAnalysis 5.0 with a signal-to-noise ratio of 5.0 for peak picking and CID was used to confirm the identity of CRMs, no charging additives were added due to sufficient signal in positive ionization mode. To reduce complex adduction for CID measurements, 0.1% of formic acid (*v*/*v*) was added prior to the acquiring of a sufficient number of scans for dissociation experiments at 4 Mw, resulting in a transient of 1.6778 s.

### 3.5. Statistical Analysis of Metabolites

The online web platform MetaboAnalyst 5.0 (https://www.metaboanalyst.ca/ (accessed on 23 March 2021)) was used for statistical analysis [38], and METLIN (https://metlin.scripps.edu/ (accessed on 23 March 2021)) was used for metabolite annotation [39]. Within MetaboAnalyst 5.0, a window of 2.5 mDa was used to bin peaks from technical and instrumental replicates by DI FT-ICR MS. Peaks within samples with greater than 80% missing values were removed, and missing values were imputed utilizing an estimated limit of detection (one-fifth the average signal). For multivariate analysis, filtering based upon standard deviation was completed, and normalization was completed to the peak area of the internal standard to remove variance from DI-ESI. Log fold changes were generated through transformation of the data. 

## 4. Conclusions

Overall, PI 567731, a SW phenotype in MGIII with a profiled QTL on chromosome 10 was profiled in drought stress field trials against Pana, a drought susceptible cultivar of *Glycine max*. Although further studies will be completed to fully profile the PI cultivar for agricultural value, multivariate analysis confirmed that the flux exhibited by drought stress detected by DI FT-ICR MS in the PI 567731 was less extensive than that exhibited by the susceptible cultivar. This response was also comparable to the control, forming predictive models for future analyses of QTLs. Statistically significant increases within the Chl content in control conditions were detected, and an expanded photosynthetic antenna within the drought affected treatment condition could account for increased photosynthetic content despite limited-maximum transpiration rates in the SW phenotype. With prior confirmation of the increased yield index and other physiological measures, profiling and observing the increased phytochemical content demonstrates the utility of this analysis in concert with physiological data for obtaining broad and focused profiled of metabolites. Furthermore, novel CRMs were probed within the analysis and were confirmed through tandem mass spectrometry to have fatty acids attached or coordinated to the porphyrin ring, with an unknown mechanism and relation into the porphyrin metabolism and photosynthesis. 

## Figures and Tables

**Table 1:** Molecular formula, theoretical mass, observed mass, and calculated mass error in ppm for the precursor and fragment ions from the CID of CRM at *m/z* 1073.70483 with 30 eV of collisional energy with argon as the collision gas.

Molecular Formula	Theoretical Mass	Observed Mass	Mass Error (ppm)
C_67_H_94_N_4_O_6_Na	1073.70656	1073.70483	1.61
C_55_H_74_N_4_O_5_Na	893.55514	893.55450	0.64
C_49_H_64_N_4_O_4_Na	795.48198	795.48019	2.21
C_35_H_36_N_4_O_5_Na	615.25779	615.25804	−0.40
C_33_H_32_N_4_O_2_Na	539.24175	539.24168	0.12

## Data Availability

Publicly available data sets were analyzed in this study. This data requires a written request with a valid email to the corresponding author, and can be found here: [https://ubuffalo-my.sharepoint.com/:f:/g/personal/twood_buffalo_edu/EmqZzPxf54xGowpsux_NCpIBN3saUrMOkfR6okV3OxqeQw?email=twood%40buffalo.edu&e=eKhNLq, accessed on 29 December 2021].

## Abbreviations

PI	plant introduction
Chl	chlorophyll
Pheo	pheophytin
CRM	chlorophyll related metabolite
SW	slow wilting
MG	maturity group
QTL	quantitative trait loci
RIL	recombinant inbred line
PS	photosystem
DI	direct infusion
GC	gas chromatography
LC	liquid chromatography
MS	mass spectrometry
FT-ICR	Fourier transform ion cyclotron resonance
PCA	principal component analysis
PLS-DA	partial least squares discriminant analysis
3D	three-dimensional
YC	young control
YDT	young drought treatment
OC	old control
ODT	old drought treatment
ANOVA	Analysis of variance
CID	Collision induced dissociation
ROS	Reactive oxygen signaling
Mw	Megaword
